# Effect of Plasma Treatment on Bamboo Fiber-Reinforced Epoxy Composites

**DOI:** 10.3390/polym16070938

**Published:** 2024-03-29

**Authors:** Pornchai Rachtanapun, Choncharoen Sawangrat, Thidarat Kanthiya, Parichat Thipchai, Kannikar Kaewapai, Jonghwan Suhr, Patnarin Worajittiphon, Nuttapol Tanadchangsaeng, Pitiwat Wattanachai, Kittisak Jantanasakulwong

**Affiliations:** 1 Faculty of Agro-Industry, Chiang Mai University, Chiang Mai 50100, Thailand; pornchai.r@cmu.ac.th; 2 Department of Industrial Engineering, Faculty of Engineering, Chiang Mai University, Chiang Mai 50200, Thailand; choncharoen@step.cmu.ac.th; 3 Office of Research Administration, Chiang Mai University, Chiang Mai 50200, Thailand; thidaratkanthiya05@gmail.com; 4 Nanoscience and Nanotechnology, Faculty of Science, Chiang Mai University, Chiang Mai 50200, Thailand; parichat_thi@cmu.ac.th; 5 Science and Technology Park (STeP), Chiang Mai University, Chiang Mai 50100, Thailand; kannikar@step.cmu.ac.th; 6 School of Mechanical Engineering, Sungkyunkwan University, 2066 Seobu-ro, Jangan-gu, Suwon-si 16419, Gyeonggi-do, Republic of Korea; 7 Department of Chemistry, Faculty of Science, Chiang Mai University, Chiang Mai 50200, Thailand; patnarin.w@cmu.ac.th; 8 College of Biomedical Engineering, Rangsit University, Pathumthani 12000, Thailand; nuttapol.t@rsu.ac.th; 9 Department of Civil Engineering, Faculty of Engineering, Chiang Mai University, Chiang Mai 50200, Thailand

**Keywords:** fiber, plasma, epoxy, reaction, bamboo

## Abstract

Bamboo cellulose fiber (BF)-reinforced epoxy (EP) composites were fabricated with BF subjected to plasma treatment using argon (Ar), oxygen (O_2_), and nitrogen (N_2_) gases. Optimal mechanical properties of the EP/BF composites were achieved with BFs subjected to 30 min of plasma treatment using Ar. This is because Ar gas improved the plasma electron density, surface polarity, and BF roughness. Flexural strength and flexural modulus increased with O_2_ plasma treatment. Scanning electron microscopy images showed that the etching of the fiber surface with Ar gas improved interfacial adhesion. The water contact angle and surface tension of the EP/BF composite improved after 10 min of Ar treatment, owing to the compatibility between the BFs and the EP matrix. The Fourier transform infrared spectroscopy results confirmed a reduction in lignin after treatment and the formation of new peaks at 1736 cm^−1^, which indicated a reaction between epoxy groups of the EP and carbon in the BF backbone. This reaction improved the compatibility, mechanical properties, and water resistance of the composites.

## 1. Introduction

Biocomposites have recently gained attention in the field of materials science as an environmentally friendly alternative to petroleum-based polymers, addressing environmental issues related to non-biodegradable petroleum polymers that impact natural organisms [1,2]. Biocomposite fibers comprise at least two components, both having different properties that contribute to the matrix and reinforcement [3]. The matrix is reinforced with natural fibers or other materials containing cellulose or agricultural waste. The development of biocomposites must consider the qualities of the components as well as their cost and environmental impact. Several natural fibers have been used to replace synthetic ones in composite materials due to their easy availability, low cost, abundance in nature, environmental friendliness, and natural biodegradability [4].

Bamboo, an abundant natural resource in Asia, is a perennial plant that can grow up to 40 m in height. It is widely used in constructing living facilities and for paper pulp in the industry [5]. Bamboo fibers (BFs) possess a cellulose composition of 36.8–54.9% [6]. BFs have been used as reinforcements in composite materials to improve their strength and alleviate the environmental impact of bamboo waste [7]. However, BFs are covered with lignin, imposing limitations in terms of the surface adhesion between the fibers and the matrix. Therefore, surface improvement is important to increase interfacial adhesion and compatibility. Various techniques, such as alkali treatment, silane coupling agent treatment, acetylation treatment, γ˗ray irradiation, plasma treatment, ultraviolet irradiation, and corona treatment, can be used to improve the surface of the fibers [8,9,10].

Plasma surface treatment involves activating and grafting polymers onto surfaces to improve various material properties, including adhesion and wettability [11]. Non-thermal or cold plasma is used for fruit applications [12]. Cold plasma presents an innovative non-thermal treatment technique with significant potential for natural fiber treatment, owing to its ecofriendly qualities. Cold plasma is applied to prevent microbial growth in food applications. The plasma treatment process provides a variety of effects, such as negative ions, positive ions, free electrons, reactive nitrogen species, reactive oxygen species, and ozone. These reactive species affect material components, surface modification, and mechanical properties. Non-thermal plasma is produced from different sources. Electric fields, heat, chemical reactions, pressure, microwaves, radio frequencies, X-rays, and electromagnetic fields are methods available to exert energy on a neutral gas [13]. Electric discharge is plasma created from an electromagnetic field. Charge carriers are induced in the electrical field and transfer energy by hitting gas particles. Electrons are oriented in the electric field while energy hits gas particles. This process creates more electrons and ions. Dielectric barrier discharge (DBD) plasma, a non-thermal or cold plasma generated by a DBD plasma generator using a high-voltage alternating current, is employed for this purpose [14]. The DBD plasma technique is an effective method, using low voltage applications in atmospheric conditions. It is used to improve the surfaces of fibers and polymers. The advantages of the DBD technique are low-pressure treatment, reproducibility, ease of control, stability, and inexpensive technology [15]. DBD plasma has received more attention due to its production of highly active species and its high electron density. Atmospheric plasma is useful to modify the surface properties of materials such as surface tension, polarity, surface roughness, and wettability. Lignin, hemicellulose, and impurity of fibers are removed by the DBD plasma technique. This approach removes compounds from cellulose fibers, increasing surface roughness and improving compatibility and adhesion between the surface and the matrix [15,16]. Plasma treatment of a natural fiber alters the structure, fiber surface, and chemical composition of the fiber. Plasma induces reactions on natural fiber surfaces by creating active sites and breaking bonds, which destroys the surface layer of natural fibers.

An epoxy (EP) is a polymer containing epoxy groups, characterized by high cross-linking and strong adhesion to reinforcing fiber surfaces. They are widely used in thermoset matrices in composites [17] due to their favorable mechanical properties, chemical resistance, shape stability, and cost effectiveness [18]. EPs have been widely applied in various industrial processes, such as coating, adhesive, and molding. Bisphenol A diglycidyl ether is a common material used to prepare EP. Amine- or amide-based hardeners are employed to create the network structure. Epoxy contains epoxy groups in the structure which react with amides, hydroxyl, and carboxylic groups. It is synthesized using a reaction of bisphenol A with epichlorohydrin [19]. Epoxy is applied to some applications due to its excellent mechanical properties, inexpensiveness, flame retardancy, and good adhesive strength. EP is a thermoset polymer with high brittleness. Reduction of crosslink density and addition of toughening agents are used to overcome the brittleness problem of EP. Toughening EP with fibers is one of the ways to improve EP properties. The addition of natural fiber into an EP matrix not only reduces the cost of EP, but also enhances its mechanical properties.

Using natural fibers to develop composites offers a promising alternative for the synthesis of materials, but faces challenges due to poor dispersion and incompatibility between the natural fibers and the matrix. Physical methods for surface modification have gained considerable attention. Plasma treatment induces physical and chemical changes in the fiber surface while preserving its original features. Moreover, it is a dry and environmentally friendly process. DBD operates at atmospheric or medium pressures, eliminating the need for vacuum equipment and allowing for the treatment of large objects on a continuous production line [14]. Previous research has shown significant improvements in surface roughness, adhesion, and shear strength after plasma treatment [20]. However, a knowledge gap exists in detailing the chemical reactions and property improvements from bamboo fiber surface treatment using plasma technology to enhance interfacial adhesion between a fiber surface and an EP matrix. The effects of DBD plasma on natural fibers within different types of gas have not been reported. The choice of gas significantly influences the ability of the fiber surface treatment to improve bonding and roughness. The generated polarity, roughness surface, and reaction are the key points to investigate for deep details of knowledge about the mechanisms in the polymer composites. Generated free radicals are expected to occur during DBD plasma treatment and form a reaction with reactive functional groups of the polymer matrix. Epoxy resin is a polymer with reactive functional epoxy groups, which can be reacted with other functional groups. The improvement of the mechanical properties of epoxy resin is a challenge addressed with toughening from the addition of some natural fibers. However, compatibility and interfacial adhesion between epoxy resin and natural fiber are poor. Therefore, the improvement of interfacial adhesion between a polymer matrix and a natural fiber using chemical reaction via the DBD plasma technique is investigated and explained in deep detail to develop a new toughening approach for epoxy resin with a natural fiber for wide application.

In this study, BFs were plasma-treated with argon (Ar), argon and oxygen (Ar+O_2_), and argon and nitrogen (Ar+N_2_) gas for 30 min to improve the fiber surface and reinforce the subsequent EP composite. Plasma treatment improved the surface and polarity of the BFs, facilitating a reaction with the EP matrix to improve the properties of the composite. The effect of the plasma treatment on the properties of EP/BF composites was investigated. The mechanical properties were evaluated based on their tensile strength, flexural strength, and flexural modulus. The morphology was examined using scanning electron microscopy (SEM), the effect of water resistance was assessed by contact angle, crystallinity was analyzed via X˗ray diffraction (XRD), and the reactions of the composites were confirmed using Fourier transform infrared (FTIR) spectroscopy.

## 2. Materials and Methods

### 2.1. Materials

BFs were sourced from South Samoeng, Chiang Mai, Thailand. The EP resin (Part A), grade A 0302, and hardener (Part B), grade A 0301, were purchased from Easy Resin, Co., Ltd., Nonthaburi, Thailand. Sodium hydroxide (NaOH) and sodium chlorite (NaClO_2_) were obtained from Merck & Co., Inc., Darmstadt, Germany.

### 2.2. Preparation of Bamboo Fibers

Bamboo trunks were peeled, cut into 2 × 6-inch pieces, and dried at 105 ± 3 °C for 12 h. The dried bamboo was ground into powder using a grinder (Grinder ML-SC5-III, Ming Lee Industrial Ltd., Hong Kong, China) and further dried at 105 ± 3 °C for 3 h. The percentage yield of the dried pulp was calculated. In alkaline treatment, 100 g of bamboo powder was mixed with NaOH 20% *w*/*v* at 80 °C, stirred at 1000 rpm for 5 h, washed with distilled water (pH 6.5–7), and filtered under vacuum. The bamboo pulp was then bleached with NaClO_2_ to remove lignin and hemicellulose. The dried pulp (100 g) was mixed with acetate buffer comprising 5.4% NaOH *w*/*v* and 150 mL of acetic acid in 1000 mL of distilled water. The mixture was boiled in 3.4% NaClO_2_ 1000 mL at 85 ± 5 °C for 3 h with stirring at 500 rpm. The final product was washed with distilled water until the pH reached 6.5–7 and then filtered under vacuum. The final cellulose product was obtained by drying the bleached pulp at 85 ± 3 °C for 12 h (bleaching repeated twice). The cellulose was sieved through a 180 µ sieve. Dielectric barrier discharge (DBD) plasma was used to treat the fiber surface. Two electrodes generated DBD plasma for etching and generated polar groups on the fiber surface. The electrodes, with RF power supply at a frequency of 13.56 MHz, were used to expose samples under the grounded electrode at a rate of 30 cm/s. The discharge gap was 1 mm. Plasma discharge power was set at 180 W (3.45 W/cm^2^). Low-frequency plasma with long wavelengths was created and provided ions via a large amount of kinetic energy. Argon (Ar), oxygen (O_2_), and (N_2_) gas were employed with rates of 8, 10, and 10 L/min, respectively. The plasma-treated bamboo powder underwent treatment with Ar, argon with oxygen (Ar+O_2_), and argon with nitrogen (Ar+N_2_) gas for 30 min [15]. All plant experiments were conducted in accordance with relevant institutional, national, and international guidelines and legislation.

### 2.3. Preparation of Composites

The EP resin and hardener were mixed in a ratio of 2:1, respectively. Untreated (BF_untr_) and plasma-treated (BF_tr_) BFs were mixed to prepare the mixed samples (EP/BF). The fibers were stirred to enable dispersion, and vacuuming was performed to remove air bubbles in the EP. The resulting mixture was cast into a silicone mold to form a bone-shaped sample and dried at 80 °C for 5 h. Details of proportions and sample names are presented in Table 1.

### 2.4. Mechanical Properties

Tensile properties were measured following the JISK˗6251˗7 standard [21] (Model MCT-1150, Tokyo, Japan). The tensile strength (TS) and elongation at break (EB) of samples were observed at a crosshead speed of 50 mm/min. The samples were prepared in a bone-shaped configuration with dimensions of size 2 mm × 5 mm × 1 mm (width × length × thickness). Ten replicates were analyzed for each sample.

### 2.5. Flexural Test

The flexural test, conducted via three-points bending analysis, aimed to determine the mechanical properties of the composite materials following the D709 standard [22], using a universal testing machine (UTM) model from H1KS, Hounsfield Test Co., Ltd., Surrey, UK. Sample sizes were prepared as 13 mm × 65 mm × 3 mm (width × length × thickness) using a force of 1 kN at room temperature. The strength of the composites was calculated using Equation (1), and flexural modulus was determined through Equation (2).

(1) $$\mathrm{Flexural}\;\mathrm{strength}\;(\sigma_{f})=\frac{3\mathrm{LF}}{2bd^{2}}$$

Here, *σ_f_* is flexural strength, F is the maximum load, L is the length of the composites, b is width, and d is thickness.

(2) $$\mathrm{Flexural}\;\mathrm{modulus}\;(E_{B})=\frac{mL^{3}}{4bd^{3}}$$

Here, *E_B_* is the flexural modulus, L is length, m is the slope of the stress-strain curve, b is width, and d is thickness. Five specimens of each sample were tested.

### 2.6. Scanning Electron Microscopy (SEM)

The morphological properties of the samples were examined at 15 kV using SEM (SEM; JSM-IT300LV, Tokyo, Japan). Sheets of the samples were created by casting them into a silicone mold at 80 °C for 5 h. Subsequently, the samples were broken in liquid nitrogen and coated with a thin layer of gold using sputtering (108 Auto/SE sputter coater; Cressington Co., Ltd., Watford, UK).

### 2.7. Contact Angle

The water contact angle was observed using drop shape analysis (DSA30E, Krüss Co., Ltd., Hamburg, Germany). Samples were prepared by casting into a silicone mold. Water droplets were applied to the sheet and recorded over 0–10 min to assess sample wettability. Five replicates were analyzed for each sample.

### 2.8. X-ray Diffraction (XRD)

XRD analysis was performed using an X-ray diffractometer (Rigaku Mini Flex, Tokyo, Japan) with Cu Kα at 40 kV. The analysis utilized a scanning rate of 2°/min in the 2Ɵ scanning mode between 2–80°.

### 2.9. Fourier Transform Infrared Spectroscopy

A Fourier transform infrared spectrometer (FTIR; Nicolet 6700, Thermo Fisher Scientific, Woodland, CA, USA) was used for FTIR analysis. Samples were obtained by casting in a silicone mold and observed in the range of 500–4000 cm^−1^, with 16 scans at 4 cm^−1^ resolution using ATR mode.

### 2.10. Statistical Analysis

Data were subjected to analysis of variance (ANOVA) to determine statistical differences, followed by multiple comparisons using Duncan’s test with SPSS software (version 17.0). Statistical significance was set at *p* < 0.05.

## 3. Results and Discussion

### 3.1. Mechanical Properties

**Figure 1:**
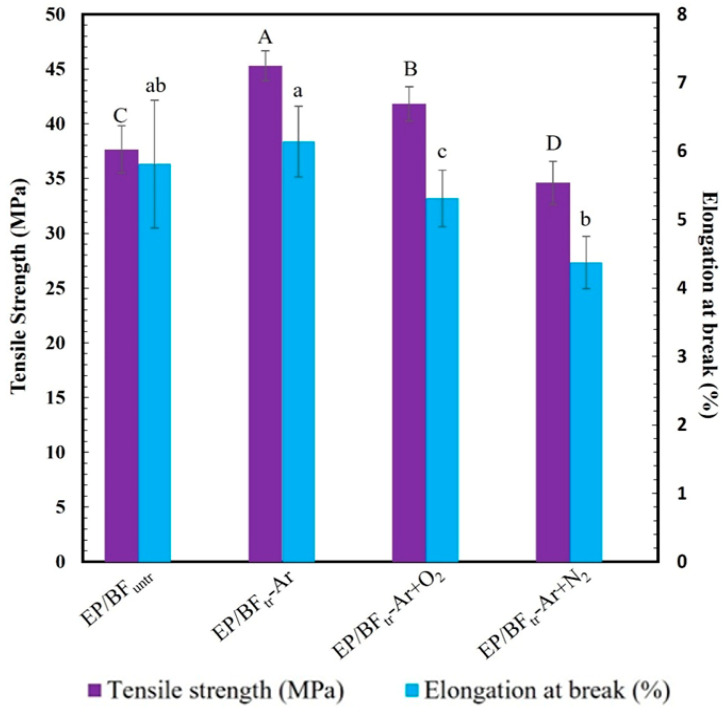
Tensile strength and elongation at break of EP/BF_untr_ and EP/BF_tr_ with Ar, Ar+O_2_, and Ar+N_2_. Mean values of the elongation at break (lowercase letters) and tensile strength (uppercase letters) differ significantly (*p* < 0.05).

Figure 1 illustrates the tensile properties of EP/BF_untr_ and EP/BF_tr_ treated with Ar, Ar+O_2_, and Ar+N_2_ for 30 min. The tensile strength and elongation at break of EP/BF_untr_ were 37.7 MPa and 5.8%, respectively. In contrast, the EP/BF_tr_ composite treated with Ar gas for 30 min exhibited higher tensile strength and elongation at break, at 45.3 MPa and 6.1%, respectively. Tensile strength and elongation at break of EP/BF_tr_ significantly decreased with Ar+O_2_ gas plasma (41.8 MPa and 5.3%) and Ar+N_2_ gas (34.6 MPa and 4.4%), respectively. The improved tensile properties of EP/BF_tr_˗Ar (45.3 MPa) were attributed to Ar gas improving the BF surface through high plasma electron density [23]. Ar gas plasma increased the surface area and roughness of the BFs, resulting in better dispersion in the EP matrix and improved interface adhesion [24]. The EP/BF_tr_ composite treated with Ar+O_2_ exhibited reduced tensile properties due to the oxidation reaction of oxygen gas, BF surface cracking, and weak adhesion of the composite [25,26]. EP/BF_tr_ composites treated with Ar+N_2_ experienced decreased tensile strength and elongation at break compared to other samples, as N_2_ gas led to BF degradation, reducing the interfacial adhesion of EP/BF [27].

### 3.2. Flexural Test

**Figure 2:**
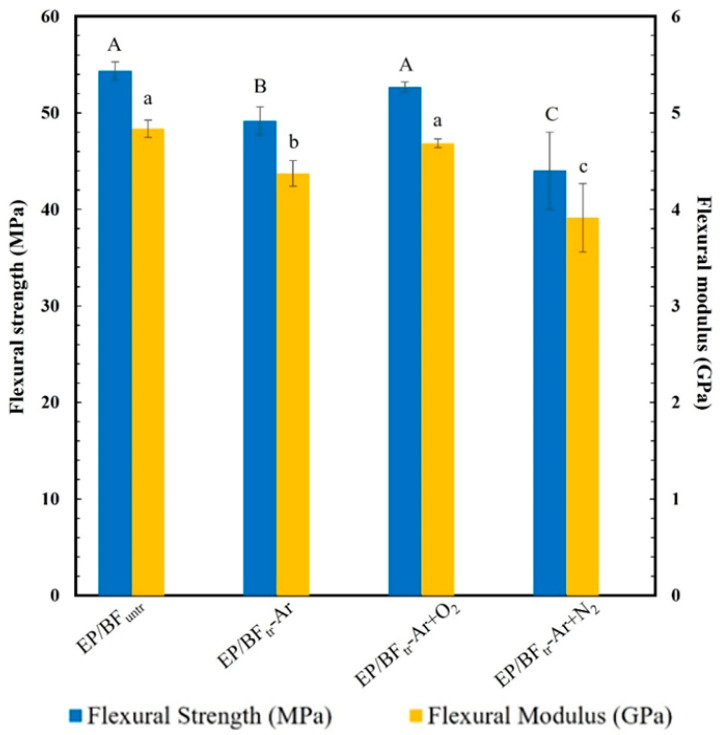
Flexural strength and flexural modulus value of EP/BF_untr_ and EP/BF_tr_ with Ar, Ar+O_2_, and Ar+N_2_. Mean values of the flexural modulus (lowercase letters) and flexural strength (uppercase letters) differ significantly (*p* < 0.05).

Figure 2 illustrates the calculated flexural properties of the EP/BF composites. The flexural strength of EP/BF_untr_ composites was 54.4 MPa, with a flexural modulus of 4.8 GPa. EP/BF treated with Ar+O_2_ showed a flexural strength of 52.7 MPa and a flexural modulus of 4.7 GPa, which did not significantly differ from those of EP/BF_untr_. However, the flexural strength (49.2 and 48.2 MPa) and modulus (4.4 and 3.9 GPa) reduced in EP/BF composites treated with Ar and Ar+N_2_ gas, respectively, due to nitrogen gas treatment inducing degradation between the EP and BFs, reducing interfacial adhesion. The EP/BFtr−Ar+O_2_ mixture exhibited the highest flexural strength, indicating the effectiveness of argon/oxygen plasma treatment, thereby improving BF polarity and bonding with the EP matrix [28]. The treatment also etched the BF surface, improving the roughness and voids in the EP/BF composites [29,30].

### 3.3. Scanning Electron Microscopy (SEM)

**Figure 3:**
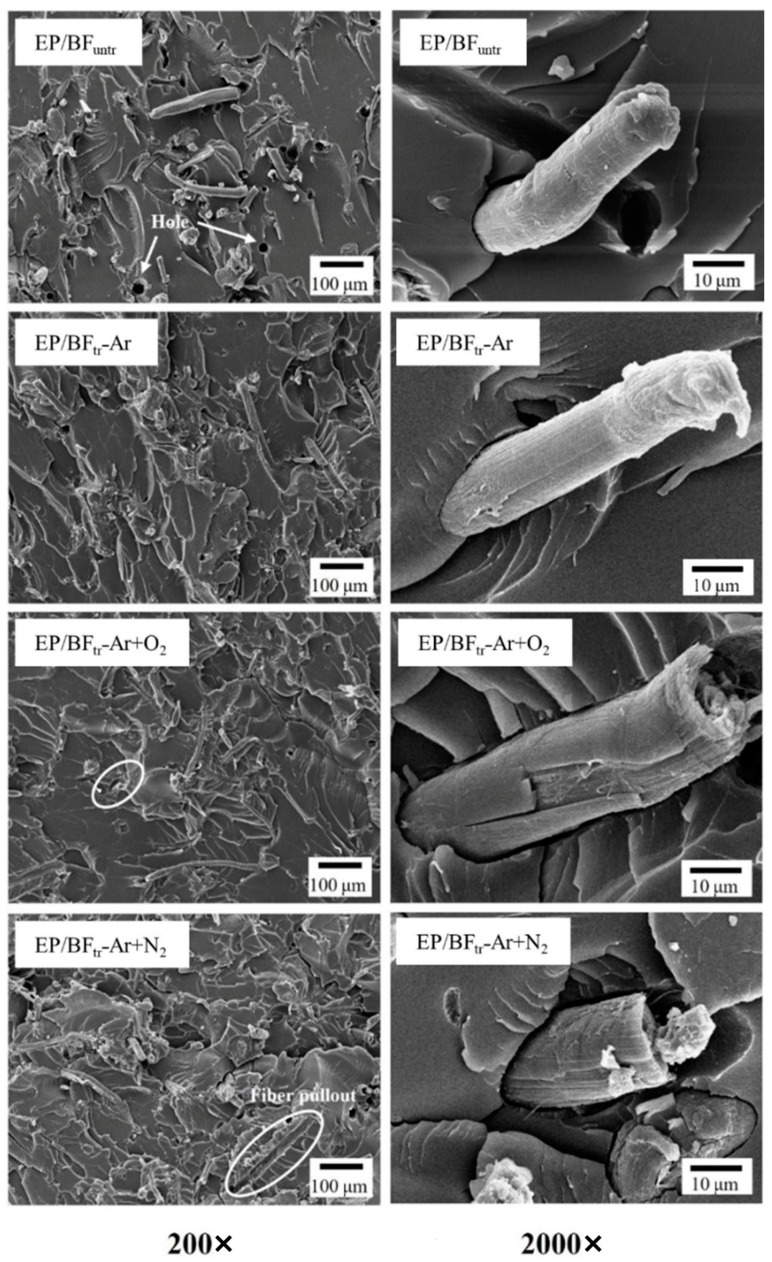
Morphology of EP/BF_untr_ and EP/BF_tr_ treated with Ar, Ar+O_2_, and Ar+N_2_ gas at 200× and 2000×.

SEM scanning was employed to examine the fractured surfaces of the composites. Figure 3 shows the morphology of the EP blend with untreated fibers and those treated with different gas plasmas treatments at 200× and 2000× magnification. EP/BF_untr_ exhibited the formation of holes on the EP surface at 200×, and fibers were pulled out at 2000×. Following plasma treatment, EP/BF_tr_−Ar showed surface improvement, with no observed fiber pull-out, indicating excellent adhesion between the EP and BFs [31]. The etching of the fiber surface with Ar gas increased BF surface roughness, improving the interfacial adhesion of EP/BF and decreasing the number of voids in the composites [32,33]. However, the interfacial bonding of the samples treated with O_2_ and N_2_ gases showed the extent of the gap between the fibers and matrix, with large holes from fiber removal in both gases. The removed BF holes on fracture surface images related to the low interfacial adhesion between fiber and epoxy matrix. Plasma treatment with Ar+O_2_ gas resulted in higher surface etching than with Ar gas due to the oxidation of O_2_ gas on the fiber surface [34,35]. O_2_ gas reduced the polarity of the fiber by formation of oxygen bonding. Oxidation by O_2_ also appeared to provide degradation around the fiber surface and decrease adhesion between the surface of the fiber and epoxy. The sample treated with Ar+N_2_ gas presented fiber pull-out and a larger number of voids than O_2_ gas, indicating fiber degradation from N_2_ gas. Low interfacial adhesion and fiber degradation decreased the mechanical properties of EP/BF_tr_−Ar+N_2_. The EP/BF_tr_−Ar sample was in the best condition to provide good morphology via the occurred reaction and high roughness of the surface of the fiber without surface degradation.

### 3.4. Contact Angle

**Figure 4:**
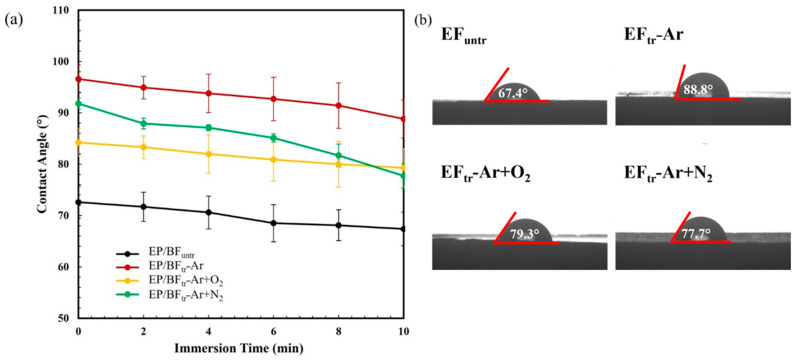
Water contact angle of EP/BF_untr_, EP/BF_tr_−Ar, EP/BF_tr_−Ar+O_2_, and EP/BF_tr_−Ar+N2; (**a**) water contact angle of samples and (**b**) image of water contact angle at 10 min.

The water droplet contact angles of the composites are shown in Figure 4a. Water droplets were dropped onto their surface and automatically recorded at 0, 2, 4, 6, 8, and 10 min. EP/BF_untr_ exhibited the lowest contact angle (67.4°) after 10 min (Figure 4b). The contact angle for EP/BF_tr_−Ar increased to 88.8° compared to EP/BF_untr_, indicating hydrophobization of the composite. Ar gas improved EP/BF compatibility and increased interfacial adhesion between BFs and the EP matrix [36]. The water contact angles of EP/BF_tr_ samples with Ar+O_2_ and Ar+N_2_ gas treatments were 79.3° and 77.7°, respectively. Plasma treatment etches the surface of the fiber, increasing roughness and the number of ions on the sample surface [37,38]. Plasma treatment with Ar+O_2_ gas resulted in high surface etching due to the oxidation of oxygen gas [26]. Poor interfacial adhesion between the fiber and EP allowed water to penetrate the interphase gap, decreasing water resistance, while the plasma treatment increased the contact angle and surface tension of the composites [39].

### 3.5. X-ray Diffraction (XRD)

**Figure 5:**
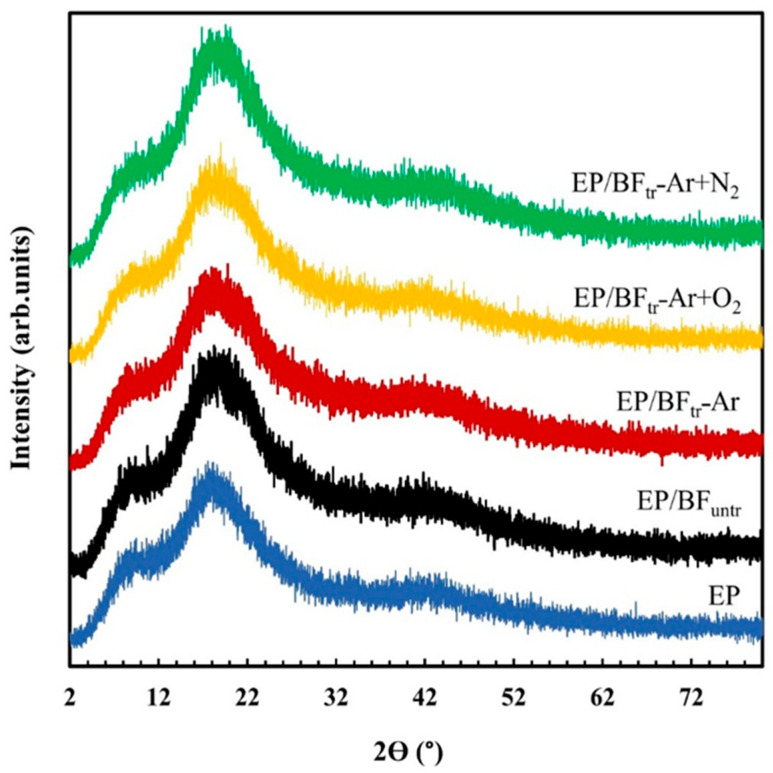
XRD pattern of EP, EP/BF_untr_, and EP/BF_tr_ treated with Ar, Ar+O_2_, and Ar+N_2_ gas.

Figure 5 shows the X˗ray patterns of the EP blend with untreated BFs and BFs plasma-treated with argon, argon/oxygen, and argon/nitrogen gas. XRD spectra of the composite showed a broad peak at 2Ɵ in the range of 5–45°, indicating the amorphous structure of the EP with peaks of 7.9°, 18.1°, and 42° [40]. Upon loading untreated and plasma-treated fibers into the EP matrix, the peaks appeared similar to those of neat EP. The degree of crystallinity of the composites was not affected by addition of both treated and untreated fibers [41], attributed to the good dispersion of BFs in the EP matrix and network structure of EP [42,43,44]. Epoxy resin forms in a reaction with a hardener to form a network structure without the formation of crystals. Crystal formation of a polymer composite is achieved by addition of a filler or nucleating agent [45]. Crystal formation of EP/BF composites was not affected with the addition of BFs due to crosslinking of EP by the reaction between epoxy groups of the EP with –NH groups of the hardener. The large molecules of plasticizer additions and polymer crosslinking prevented the movement of polymer chains to form crystals [21]. This network structure of epoxy resin provides amorphous structure without changing the XRD peak.

### 3.6. Fourier Transform Infrared Spectroscopy

**Figure 6:**
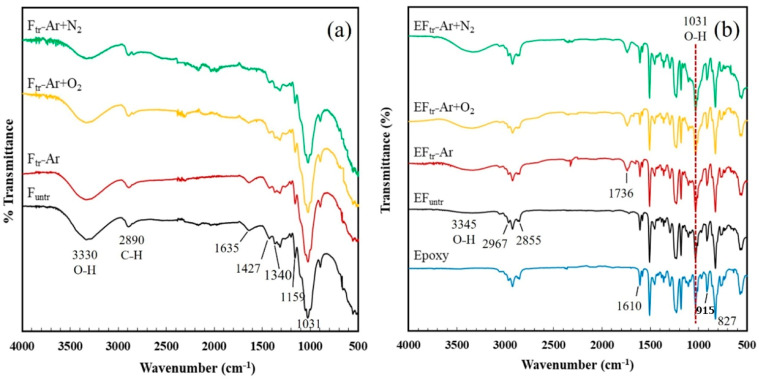
FT-IR spectra of (**a**) untreated BFs and BFs plasma-treated with three gases and (**b**) EP/BF_untr_, EP/BF_tr_ plasma-treated with Ar; EP/BF_tr_−Ar, Ar+O_2_; EP/BF_tr_−Ar+O_2_ and Ar+N_2_; EP/BFtr−Ar+N_2_.

Figure 6a shows the FTIR spectra of untreated and plasma-treated fibers. The spectra showed transmission bands at 3330 cm^−1^, corresponding to the hydroxyl (OH) stretching vibration of hydrogen-bonded fibers [46,47]. The peak around 2890 cm^−1^ was associated with the C–H stretching vibration of cellulose fiber [48,49]. The band at 1635 cm^−1^ was attributed to the aromatic nature of lignin [50,51,52]. Peaks at 1427, 1340, 1159, and 1031 cm^−1^ correspond to CH_2_, CH_3_ bending vibration (methoxyl group in lignin), and C–O stretching of cellulose fibers, respectively [53,54,55]. Following plasma treatment, the intensity of the band at 1635 cm^−1^ decreased due to the reduced lignin components [56]. Figure 6b shows the FTIR spectra of untreated and plasma-treated EP/BF composites. EP exhibited peaks at 2926 and 2858 cm^−1^, for asymmetric and symmetric CH_2_ and CH_3_, respectively [57]. Peaks at 1456, 1508, 1580, and 1606 cm^−1^ corresponded to C–C stretching vibration of the aromatic ring of EP [58], while characteristic peaks at 915 cm^−1^ and 1610 cm^−1^ signified stretching epoxide ring vibration [59,60]. In the EP/BF_tr_ composites, an increase in peak intensity at 3200–3600 cm^−1^ was observed, attributed to O–H stretching of hydroxyl groups. A strong band at 1033 cm^−1^ indicated C–O and C–OH stretching vibration of cellulose [55]. Plasma with Ar, O_2_, and N_2_ generated the polar groups of C–C^−^, C–O^−^, and –COO^−^ by removing hydrogen bonding. These functional groups can form reactions with epoxy groups. The occurred reactions provided new strong covalent bonds, which increased interfacial adhesion between fiber surface with epoxy matrix. A new peak at 1736 cm^−1^ was observed due to plasma treatment, generating new functional polar groups on the fiber surface [61]. The reaction indicated that–COO^−^ groups on the structure of the fiber reacted with epoxy groups of the epoxy. It was suggested that these bonds at the fiber surface reacted with the epoxy groups of the EP, resulting in strong interfacial adhesion and improving properties [62,63,64].

## 4. Conclusions

A BF-reinforced EP composite was successfully developed using plasma treatment. The influence of plasma-treated bamboo fibers on the properties of BF reinforcement and EP was investigated. Tensile strength significantly improved from 37 to 45 MPa after 30 min of Ar gas plasma treatment, attributed to lignin and impurity removal and increased BF surface roughness. The flexural strength and flexural modulus of the EP/BF composite increased with Ar+O_2_ plasma treatment, improving polarity and surface roughness, thereby promoting connection between the EP and BF surfaces. SEM images showed fiber pull-out in the EP/BF_untr_ sample, and a small gap between the BFs and EP in EP/BF_tr_. Argon gas treatment increased the water contact angle, due to the enhanced BF surface roughness from plasma etching, and improved compatibility between the BFs and EP. XRD spectra presented the amorphous structure of the EP in the composites, unaffected by BF loading. FTIR analysis showed lignin alterations after plasma treatment, with a new peak at 1736 cm^−1^, indicating the generation of new functional polar groups on the fiber surface. Strong bonding resulted in robust interfacial adhesion between the EP and BFs. The reaction between epoxy groups of EP and new C–O groups of the BFs improved the mechanical properties and water resistance of the composites. Such composites hold potential value in coating, packaging, agricultural, and medical applications.

## Figures and Tables

**Table 1:** Composition and sample code of epoxy composite samples (%wt/wt).

Samples	Gas			Epoxy: Hardener (2:1)	Fiber
	Argon	Oxygen	Nitrogen	(%)	(%)
EP/BF_untr_				95	5
EP/BF_tr_˗Ar 30 min	✓			95	5
EP/BF_tr_˗Ar+O_2_ 30 min	✓	✓		95	5
EP/BF_tr_˗Ar+N_2_ 30 min	✓		✓	95	5

## Data Availability

The data presented in this study are available upon request from the corresponding author.

## Abbreviations and Symbols

ER, epoxy resin; BFs-Bamboo Fibers; DBD, Dielectric barrier discharge; FTIR, Fourier-transform infrared spectroscopy; SEM, scanning electron microscopy; XRD, X-ray diffraction.
